# Investigation of urinary volatile organic metabolites as potential cancer biomarkers by solid-phase microextraction in combination with gas chromatography-mass spectrometry

**DOI:** 10.1038/bjc.2011.437

**Published:** 2011-11-15

**Authors:** C L Silva, M Passos, J S Câmara

**Affiliations:** 1 CQM/UMa – Centro de Química da Madeira, Centro de Ciências Exactas e da Engenharia, Universidade da Madeira, Campus Universitário da Penteada, Funchal 9000-390, Portugal; 2 Hospital Dr Nélio Mendonça, Avda. Luís de Camões, Funchal 9000, Portugal

**Keywords:** urine, volatile organic metabolites, biomarkers, HS-SPME/GC-qMS

## Abstract

**Background:**

Non-invasive diagnostic strategies aimed at identifying biomarkers of cancer are of great interest for early cancer detection. Urine is potentially a rich source of volatile organic metabolites (VOMs) that can be used as potential cancer biomarkers. Our aim was to develop a generally reliable, rapid, sensitive, and robust analytical method for screening large numbers of urine samples, resulting in a broad spectrum of native VOMs, as a tool to evaluate the potential of these metabolites in the early diagnosis of cancer.

**Methods:**

To investigate urinary volatile metabolites as potential cancer biomarkers, urine samples from 33 cancer patients (oncological group: 14 leukaemia, 12 colorectal and 7 lymphoma) and 21 healthy (control group, cancer-free) individuals were qualitatively and quantitatively analysed. Dynamic solid-phase microextraction in headspace mode (dHS-SPME) using a carboxen-polydimethylsiloxane (CAR/PDMS) sorbent in combination with GC-qMS-based metabolomics was applied to isolate and identify the volatile metabolites. This method provides a potential non-invasive method for early cancer diagnosis as a first approach. To fulfil this objective, three important dHS-SPME experimental parameters that influence extraction efficiency (fibre coating, extraction time and temperature of sampling) were optimised using a univariate optimisation design. The highest extraction efficiency was obtained when sampling was performed at 50°C for 60 min using samples with high ionic strengths (17% sodium chloride, w v^−1^) and under agitation.

**Results:**

A total of 82 volatile metabolites belonging to distinct chemical classes were identified in the control and oncological groups. Benzene derivatives, terpenoids and phenols were the most common classes for the oncological group, whereas ketones and sulphur compounds were the main classes that were isolated from the urine headspace of healthy subjects. The results demonstrate that compound concentrations were dramatically different between cancer patients and healthy volunteers. The positive rates of 16 patients among the 82 identified were found to be statistically different (*P*<0.05). A significant increase in the peak area of 2-methyl-3-phenyl-2-propenal, *p*-cymene, anisole, 4-methyl-phenol and 1,2-dihydro-1,1,6-trimethyl-naphthalene in cancer patients was observed. On average, statistically significant lower abundances of dimethyl disulphide were found in cancer patients.

**Conclusions:**

Gas chromatographic peak areas were submitted to multivariate analysis (principal component analysis and supervised linear discriminant analysis) to visualise clusters within cases and to detect the volatile metabolites that are able to differentiate cancer patients from healthy individuals. Very good discrimination within cancer groups and between cancer and control groups was achieved.

Cancer is characterised by abnormal growth and development of normal cells beyond their natural boundaries. Despite global efforts to limit the incidence of this disease, cancer has become the leading cause of death in the last 50 years. Specifically, breast cancer is the most common malignancy and the second most common cause of cancer-related mortality in women. Prostate cancer is the most common solid organ malignancy diagnosed in men in Europe and the United States of America and is the second most frequent cause of cancer-related death in men (Ullah and Aatif, 2009; Jemal *et al*, 2010). Furthermore, after cardiovascular diseases, cancer is the second cause of death among the global population (Infante *et al*, 1994; Weisburger and Williams, 2000; Oliveira *et al*, 2007).

The factors that are responsible for cancer development are classified as exogenous and endogenous (Oliveira *et al*, 2007). The first group includes nutritional habits (food preservation and preparation), socio-economic status, lifestyle, physical agents (ionising and non-ionising radiation), chemical compounds (natural and synthetic) and biological agents (*Helicobacter pylori*, Epstein–Barr virus, human T lymphotropic viruses I and II, human papilloma virus and the hepatitis B virus, as well as parasites, such as *Schistosoma haemotobium*, *Clonorchis sinensis* and *Opisthorchis vivarium*) (Minamoto *et al*, 2000; Weisburger and Williams, 2000). Unhealthy lifestyle habits such as excess alcohol consumption, the inhalation of tobacco and related products, the ingestion of certain foods and their contamination by mycotoxins are responsible for higher incidences of certain types of neoplasias in a number of population groups (Weisburger and Williams, 2000; Oliveira *et al*, 2007). Endogenous factors include immune system damage and inflammation caused by uncertain aetiology (e.g., ulcerative colitis and pancreatitis), genetic makeup, age, endocrine balance and physiological condition (Barrett and Anderson, 1993; Infante *et al*, 1994).

The management of high-risk cancers requires diagnosis at an early stage, which specifies the need for specific and sensitive biomarkers. Sometimes, certain molecules are differentially expressed in cancer cells relative to their normal counterparts, and their altered levels can be measured to establish a correlation with the diseased state (Cassiday, 2006; Jemal *et al*, 2010). With the development of high-throughput techniques for biomarker discovery, the field of cancer biomarkers has rapidly expanded (Lauridsen *et al*, 2007; Gramolini *et al*, 2008; Gaspar and Lopes, 2009; Jiang and Ma, 2009; Matsumura *et al*, 2010). Current biomarker candidates from blood, sputum and urine include many classes of molecules, such as proteins, tumour-specific antigens, anti-tumour antibodies, cell type-specific peptides, various metabolic products and epigenetic phenomena (e.g., hyper-methylated DNA, RNA and specific gene expression) (Greenberg and Lee, 2007; Matsumura *et al*, 2010); however, according to Matsumura *et al* (2010), no biomarker identified to date has been shown to have adequate sensitivity, specificity and reproducibility so as to be considered sufficient for use in the detection and monitoring of lung cancer development.

One promising class of biomarkers for cancer could be low-molecular-weight volatile organic metabolites (VOMs) (Matsumura *et al*, 2010). The ‘volatile hypothesis’, which was initially postulated for lung cancer, has led to a number of studies examining the utility of analysing these metabolites in the exhaled breath of animals (such as dogs) or in sophisticated biochemical techniques (McCulloch *et al*, 2006; Belda-Iniesta *et al*, 2007; Song *et al*, 2010). Thousands of VOMs are present in trace amounts in human breath, and different studies have shown that VOM profiles of patients with lung cancer can be discriminated from those of healthy subjects (Phillips *et al*, 2003, 2006; Mazzone, 2008; Yung, 2010). A recent study from the Chen group using solid-phase micro-extraction (SPME) followed by gas chromatography (GC) has demonstrated that 1-butanol and 3-hydroxy-2-butanone were found at significantly higher concentrations in the breath of lung cancer patients in comparison with the controls (Chen *et al*, 2007).

To date, current screening trials have primarily focused on imaging modalities, such as computer tomography, magnetic resonance imaging, endoscopy and ultrasonography, together with clinical analysis; however, these methods are time-consuming and unpleasant for patients and require skilled medical staff and expensive devices. Over the past few years, however, non-imaging methods have been investigated.

Urine and breath analysis for the routine monitoring of metabolic disorders has attracted a considerable amount of scientific interest, especially because their sampling is non-invasive. These types of analyses are totally painless and agreeable to patients and can be performed as often as needed.

Apart from providing the possibility of non-invasive diagnostic testing, urine has other advantages over breath, serum or cerebrospinal fluid (Deng *et al*, 2004b; Xue *et al*, 2008). Most important, many metabolites occur in urine at nearly the same concentrations as in the plasma, whereas the total volatile metabolites in the plasma are relatively low (μg–ng l^−1^). Thus, the relative enrichment of volatile components makes urine an attractive target for a volatile metabolomic profiling approach. Urinary metabolomic studies have already been applied to the breast (Chen *et al*, 2009; Henneges *et al*, 2009), lung (Carrola *et al*, 2011), prostate (Chikkaveeraiah *et al*, 2009; Sreekumar *et al*, 2009; Jentzmik *et al*, 2010), colorectal (Ma *et al*, 2009; Qiu *et al*, 2010) and liver cancers (Chen *et al*, 2009).

In recent years, there has been an enormous effort to develop specific and sensitive biomarkers for the precise and accurate screening, diagnosis, prognosis and monitoring of high-risk cancer to assist with therapeutic decisions. Different methods have thus been developed to analyse volatile metabolites and compare them in healthy subjects and cancer patients, such as chemical interaction, adsorptive binding, cold trapping and supercritical fluid extraction. The most successful methods in this field are SPME and the recently developed multi-bed sorption trap (Giardina and Olesik, 2003; Prado *et al*, 2003; Yu *et al*, 2009). This technique was developed by Pawliszyn in late 1989 as a new pre-concentration technology in which a fused coated silica fibre is used as the stationary phase (Ouyang *et al*, 2005; Song *et al*, 2010). This methodology presents several advantages over conventional solvent extraction procedures: SPME is rapid, easy to use, solvent free and sensitive, and does not require any concentration step before analysis, preventing artefacts (Musteata and Pawliszyn, 2007).

Therefore, in this study, we tested the potentialities of a simple and solvent-free miniaturised high-throughput analytical methodology without sample derivatisation based on dynamic solid-phase microextraction in headspace mode (dHS-SPME) in combination with GC-qMS. This technique was used for the metabolomic analysis of urine samples that were obtained from clinically diagnosed patients with leukaemia, colorectal and lymphoma cancers, and from healthy controls (cancer-free) to provide comprehensive information on volatile metabolites as potential cancer biomarkers.

A comparative analysis of the urine metabolome between cancer patients and normal controls was carried out. This study presents some preliminary results in a small group of cancer patients in comparison with the controls. Six different stationary phases, polydimethylsiloxane (PDMS), polyacrylate (PA), divinylbenzene/carboxen/PDMS (DVB/CAR/PDMS), carbowax/DVB (CW/DVB), CAR/PDMS and PDMS/DVB, providing specificity for a wide range of polar and non-polar volatile compounds, were compared in terms of extraction efficiency and sensitivity. Factors that might affect the dHS-SPME procedure, such as extraction time and extraction temperature, were also investigated to determine the analytical performance of the selected fibre.

To test the applicability of our method, a total of 54 urine samples (healthy individuals, *n*=21 and cancer patients, *n*=33) were collected in the Hospital Dr Nélio Mendonça (Haemato-Oncology Unit) and analysed. Multivariate statistical methods were used to gain insight into the metabolomic differences between healthy and patient cases and find related volatile metabolites that could be associated with a unique type of cancer (e.g., leukaemia, colorectal, lymphoma). This identification is indispensable for future work on the biochemical sources of these compounds and their metabolic pathways.

## Materials and Methods

### Reagents and materials

Sodium chloride (NaCl) and hydrogen chloride (HCl) were purchased from Panreac (Barcelona, Spain) and Sigma-Aldrich (St Louis, MO, USA), respectively. Ultra-pure water from a Milli-Q system (Millipore, Bedford, MA, USA) with a conductivity of 18 MΩ was used throughout. Helium at a purity of 99.999% (Air Liquide, Lisbon, Portugal) was utilised as the GC carrier gas. A Cimarec digital stirring hot plate was supplied by Thermo Scientific (Waltham, MA, USA). The SPME holder for manual sampling of SPME fibres and glass vials was purchased from Supelco (Bellefonte, PA, USA). The SPME fibres were conditioned as recommended by the manufacturer, but below the maximum recommended temperature before their first use. Before the first daily analysis, the fibres were conditioned for 5 min at the operating temperature of the GC injector port and the blank level was checked. The analyses were performed in triplicate.

### Subjects and sample collection

The subjects were divided into four groups: normal controls and cancer patients with leukaemia, colorectal and lymphoma (Table 1). Normal controls (*n*=21, age=44.2±10.3 (range 28–60) years, 18 male and 3 female) volunteered and were eligible to participate in the study if they were 18 years of age or older and had no previously diagnosed cancer.

The subjects were selected from among the blood donors at the Hospital Dr Nélio Mendonça (Funchal, Portugal). Urine samples were collected at the Blood Bank of Funchal Hospital. In all, 33 urine specimens from patients with different cancer pathologies (oncological group) were collected at the Unit of Haematology–Oncology at the same institution. Leukaemia (*n*=14, age=50.1±12.4 (40–74) years, 6 male and 8 female), colorectal cancer (*n*=11, age=62.0±11.7 (49–78) years, 8 male and 3 female) and lymphoma (*n*=7, age=42.0±19.1 (18–68) years, 6 male and 1 female) patients were identified using specific examinations. As can be observed from these data, the groups were small and not balanced. Each individual (either patient or healthy volunteer) provided a sample of morning urine (after overnight fasting) in a 20 ml sterile PVC container. The samples were frozen at −80°C and retained until needed for the experiments. Before extraction, the pH values of the 25 ml urine samples were adjusted to 1–2.

All subjects signed an informed consent to participate in the study, and the research was approved by the Ethics Committee of Funchal Hospital.

### dHS-SPME procedure

The development of a suitable dHS-SPME method to establish a urinary volatile metabolomic profile from cancer patients and healthy volunteers involved the selection and optimisation of multiple parameters that influenced extraction. In particular, the nature of the adsorptive phase, the sampling temperature and the extraction time required to achieve equilibration between the analytes and the fibre were considered by applying a univariate experimental design.

Fibre selection was performed by testing and comparing the extraction efficiency of six SPME fibres (Supelco) to different stationary phases and various film thicknesses, including PDMS (100 *μ*m), PA (85 *μ*m), DVB/CAR/PDMS (50/30 *μ*m), CAR/PDMS (75 *μ*m), CW/DVB (70 *μ*m) and PDMS/DVB (65 *μ*m). A urine sample from a normal subject was used as the matrix for the optimisation of the dHS-SPME parameters. Frozen urine samples were completely thawed at room temperature before use.

For dHS-SPME optimisation, 4 ml aliquots of urine samples that had been adjusted to a pH of 1–2 with 50 ml of 5.0 M HCl were placed in an 8-ml headspace glass vial. After the addition of 0.8 g of NaCl and stirring (0.5 × 0.1-mm bar) at 800 r.p.m., the vial was capped with a Teflon (PTFE) septum and an aluminium cap (Chromacol, Hertfordshire, UK).

The addition of salt greatly increased the extraction efficiency for many of the metabolites, particularly the polar ones. The presence of salt can influence adsorption in two ways: by changing the properties of the phase boundary and decreasing the solubility of hydrophilic metabolites in the aqueous phase (salting-out effect). This salting-out effect is widely used to increase the sensitivities of analytical methodologies.

The sample vial was placed in a thermostat bath that was adjusted to 50.0±0.1°C and then the SPME fibre was inserted into the headspace for 60 min. After sampling, the SPME fibre was withdrawn into the needle, removed from the vial and inserted into the injector port (250°C) of the GC-qMS system for 6 min, wherein the metabolites were thermally desorbed and transferred directly to the analytical column. Each sample was analysed in triplicate. Blanks, which corresponded to analyses of coating fibres that were not submitted to any extraction procedure, were run between sets of three analyses. A CAR/PDMS fibre was used to investigate the extraction temperature and time. Extraction was performed at temperatures of 30°C, 50°C and 60°C for 30, 45, 60 and 75 min with a stirring rate of 800 r.p.m. The optimum conditions were determined by the sum of the peak areas obtained under each parameter, number of extracted metabolites and reproducibility.

### GC-qMS analysis

The SPME fibre with absorbed/adsorbed (depended on the fibre coating) volatile metabolites was inserted into the injection port of an Agilent Technologies 6890N Network gas chromatograph system (Palo Alto, CA, USA), and metabolites were desorbed for 6 min at 250°C. The gas chromatograph was equipped with a 30 m × 0.25 mm ID × 0.25 *μ*m film thickness BP-20 (SGE, Dortmund, Germany) fused silica capillary column and interfaced with an Agilent 5975 quadrupole inert mass selective detector. We employed the following chromatographic protocol for separation before the MS analyses: 35°C for 2 min, and then programmed at 2.5°C min^−1^ to 220°C with a 5-min hold at this final temperature, for a total GC run time of 77 min. The column flow was maintained constant at 1 ml min^−1^ using He (Helium N60, Air Liquide) as the carrier gas. The injection port was operated in the splitless mode and held at 250°C.

For the 5975MS system, the operating temperatures of the transfer line, quadrupole and ionisation source were 270, 150 and 230°C, respectively. Electron impact mass spectra were recorded at a 70 eV ionisation voltage and the ionisation current was 10 *μ*A. The acquisitions were performed in Scan mode (30–300 *m/z*), and the electron multiplier was set to the autotune procedure. Metabolite identification was accomplished through manual interpretation of spectra and matching against the Agilent MS ChemStation Software (Palo Alto, CA, USA) or commercially available standard samples, when available. This software was equipped with an NIST05 mass spectral library with a similarity threshold of >80%. A series of C_8_–C_20_ *n*-alkanes were analysed using the same methodology (dHS-SPME_CAR/PDMS_/GC-qMS) to establish the retention indices and to confirm the identity of the metabolites via comparison with the literature.

### Statistical analysis

Data statistical analysis was performed using the SPSS 17.0 package for Windows (SPSS Inc., Chicago, IL, USA). Significant differences among the groups were assessed with a one-way analysis of variance (ANOVA). The least square difference (LSD) test (*P*-value <0.05) was used to compare the means. Principal component analysis (PCA) was also applied to the analysed groups to verify the distribution of the variables for the referred groups.

## Results and discussion

The sequence followed in this study consisted of two steps. The first focused on obtaining the best experimental conditions to extract volatile metabolites from the urine of healthy and cancer volunteers using dHS-SPME. In the second step, an objective comparison of the metabolomic patterns found in the urine from cancer patients and healthy volunteers was established in terms of qualitative (identification by comparing the MS spectrum with the *kóvats index*) and semiquantitative (peak area ratio) differences under optimised conditions. Different profiles for healthy people and cancer patients were successfully recognised. Among other compound classes, aldehydes, ketones, terpenoids, acids, furans, volatile phenols, benzene derivatives, sulphur-containing compounds and naphthalenes were identified.

### Optimisation of the dHS-SPME parameters

The optimisation of the different parameters involved in HS-SPME was performed by choosing the conditions that achieved the maximum response in terms of metabolite peak area, number of detected metabolites and reproducibility.

### Fibre selection

**Figure 1:**
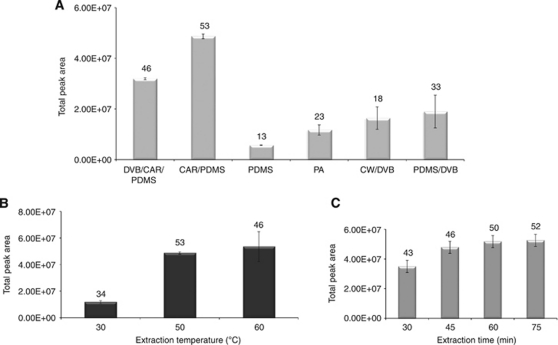
Optimisation of the solid-phase micro-extraction (SPME) influencing extraction parameters: (**A**) effect of fibre coatings (60 min of extraction time at 50°C); (**B**) effect of extraction temperature (fibre: 75 *μ*m carboxen-polydimethylsiloxane (CAR/PDMS); extraction time: 60 min); and (**C**) influence of the extraction time (fibre: 75 *μ*m CAR/PDMS; extraction temperature: 50°C) on SPME extraction efficiency of urinary volatile metabolites in a healthy individual. All assays were carried out with continuous stirring (800 r.p.m.). Thermal desorption of metabolites were performed at 250°C for 6 min. Numbers above the bars represent the total number of metabolites identified.

As far as selection of the SPME fibre was concerned, the nature of the metabolites influenced which SPME fibre was chosen. Therefore, six SPME fibres were tested to select the most appropriate to isolate volatile metabolites from urine. The results of the relative extraction efficiency for the tested fibres are shown in Figure 1A.

By comparing all of the tested fibres in terms of chromatographic areas, the number of identified metabolites and relative standard deviation (r.s.d.), the best efficiency was obtained using a CAR/PDMS coating, whereas the lowest efficiency was obtained using the PDMS and PA fibres. Each extraction was performed in triplicate and the repeatability (% r.s.d.) was lower than 20%. Thus, the CAR/PDMS fibre (Figure 1A) was chosen as the SPME fibre for the remaining optimisation studies.

### Extraction temperature and time

Using an extraction time of 60 min with 4 ml of the same urine sample, the effect of extraction temperature at 30, 50 and 60°C on SPME extraction efficiency was investigated. The volatile metabolites were extracted under acidic (pH 1–2) conditions using only the CAR/PDMS fibre. The plot of the peak areas *vs* extraction temperatures is shown in Figure 1B.

Temperature will substantially affect the diffusion rates of VOMs. Raising the temperature progressively from 30 to 50°C increased the number of extracted metabolites that were identified. Although there was a slight increase in the number of metabolites that were identified at 60°C (2 more), the r.s.d. obtained therein was higher than those for the other investigated temperatures. At high temperatures (above 50°C), there is a probable degradation of the sample; hence, 50°C was used for the remainder of the study. As outlined in Figure 1B, the temperature was fixed at 50°C for the extraction of urinary volatile metabolites from healthy volunteers and cancer patients.

The influence of extraction time on the efficiency of the SPME process was investigated by exposing the SPME fibre to the urine headspace at 50°C for 30, 45, 60 and 75 min. Sorption time profiles for volatile metabolites indicated that a sampling time of >45 min was necessary to reach equilibrium. When the equilibrium was not reached, an alternative methodology was to develop the extraction under non-equilibrium conditions, which require shorter extraction times. Figure 1C demonstrates that the equilibrium between the samples and fibre was established in 60 min. With additional extraction time, there was no obvious increase in the peak area. On the basis of the results, 60 min was chosen as the extraction time for further analysis.

### Characterisation and comparative analysis of urinary volatile metabolites

**Figure 2:**
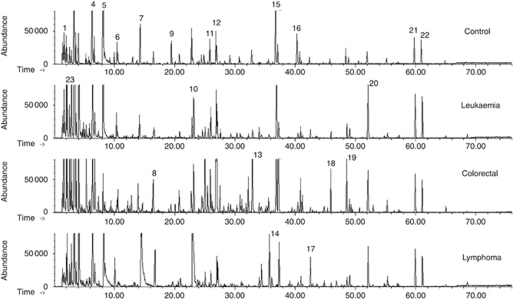
A typical urinary gas chromatograph quadrupole mass spectrometer (GC-qMS)-based metabolomics profile (fingerprint signals) of cancer patients (leukaemia, colorectal and lymphoma) contrasted with a healthy volunteer (control group). Extraction was performed using a carboxen-polydimethylsiloxane (CAR/PDMS) coating exposed to the headspace vapour for 60 min at 50°C. Peak identification: (1) methanethiol; (2) acetone; (3) 2-pentanone; (4) dimethyl disulphide; (5) 4-heptanone; (6) 4-methyl-2-pentanol (IS); (7) *γ*-terpinene; (8) 2-methoxythiophene; (9) dimethyl trisulphide; (10) linalool oxide; (11) 2-ethyl-1-hexanol; (12) vitispirane I; (13) menthol; (14) 4,7-dimethyl-benzofuran; (15) 1,2-dihydro-1,1,6-trimethyl-naphthalene; (16) *β*-damascenone; (17) *p*-cimenol; (18) 3,4-dimethyl-benzaldehyde; (19) 1-ethyl-3,5-diisopropyl-benzene; (20) 4-methyl-phenol; (21) *p*-tert-butyl-phenol; and (22) 2,4-bis(1,1-dimethylethyl)-phenol.

We next characterised the nature of the chemical variation in the collected urine samples to distinguish individuals with tumours from those without by analysing urinary volatile metabolites using solid-phase microextraction in combination with GC coupled to mass spectrometry. According to the typical GC-qMS total ion chromatograms (TICs) depicted in Figure 2, a large and diverse set of metabolites can be distinguished in the urine obtained from healthy people (control group) and that obtained from cancer patients (leukaemia, colorectal and lymphoma).

**Figure 3:**
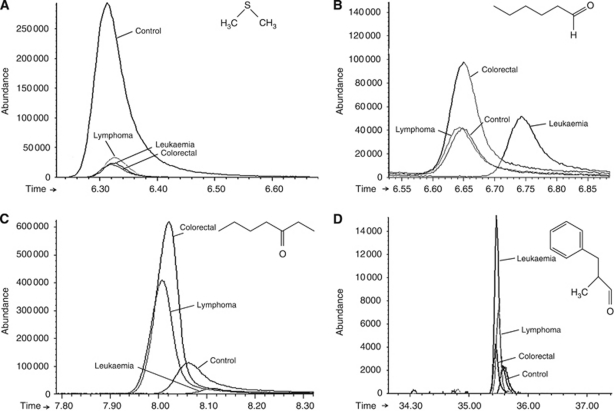
Comparison between healthy and oncological group of four illustrative metabolites selected from 82 peaks analysed. Enlarged part of the chromatograms of Figure 2 with peaks representing: (**A**) dimethyl disulphide (ion 94); (**B**) hexanal (ion 44); (**C**) 3-heptanone (ion 57); and (**D**) 2-methyl-3-phenyl-2-propenal (ion 145).

Different urinary GC-qMS profiles for healthy people and cancer patients were able to be recognised. In all, 82 of the identified volatile metabolites that were found in both the cancer and the healthy urine samples included a variety of chemical structures and were identified to be involved in multiple biological functions (e.g., pheromonal communication for 2-heptanone; Deng *et al* (2004a)). Some metabolites that have previously been reported to appear in human urine (dimethyl disulphide, methanethiol and 2-methylbutanoic acid) (Henneges *et al*, 2009) were also identified. The enlarged peaks in the TICs of some of the significant metabolites are depicted in Figure 3, and these facilitate the differentiation of metabolomic profiles.

The peak area ranges (minimum, maximum and median values) of the urinary volatile metabolites that were found in the cancer patients and healthy subjects are summarised in Supplementary Table 1. Identification was performed using a NIST05 library through comparison of fragmentation patterns with a standard mass chromatogram and verified by reference compounds, where available.

The metabolomic origin and physiological function of most of the VOMs are still not known. Their origins lie in a variety of endogenous biochemical pathways and exogenous sources (environmental, unhealthy lifestyle habits, biological agents); however, the chemical pathways of generation have not yet been explained. Some of the endogenous markers were derived from the mevalonic acid pathway of cholesterol synthesis (e.g., unsaturated hydrocarbons like isoprene), from glucose metabolism (e.g., acetone) and from the oxygen free radical-mediated lipid peroxidation of fatty acids (e.g., aldehydes and linear and branched saturated hydrocarbons). The source of the VOMs that was identified to derive from naphthalene is not yet known; they may be the degradation products of steroids. Further research would be required to determine which of these metabolites are of tumour origin and which originate from normal metabolic processes, as well as which are down- or upregulated by tumour growth.

**Figure 4:**
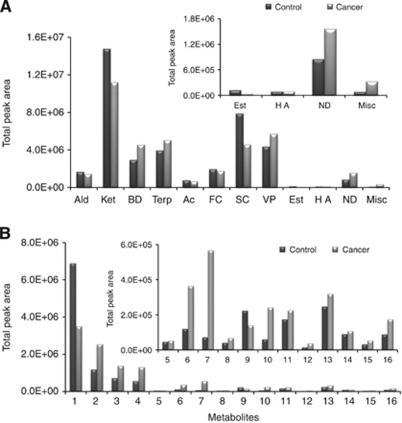
Average levels of metabolites excreted in urine samples from normal subjects (21) and cancer patients (31). (**A**) Chemical families identified in oncological and control groups (Ald – aldehydes; Ket – ketones; BD – benzene derivates; Terp – terpenoids; Ac – acids; FC – furan compounds; SC – sulphur compounds; VP – volatile phenols; Est – esters; HA – higher alcohols; ND – naphthalene derivates; Misc – miscellaneous). (**B**) Average areas for statistically significant metabolites identified in cancer patients. Numbered bars correspond to: (1) hexanal; (2) heptanal; (3) 2-methyl-3-phenyl-2-propenal; (4) 3-heptanone; (5) 1,2,4-trimethylbenzene; (6) *p*-cymene; (7) anisole; (8) *γ*-terpinene; (9) bornylene; (10) dimethyl disulphide; (11) 2-methoxythiophene; (12) 4-methyl-phenol; (13) 1-octanol; (14) 1,2-dihydro-1,1,6-trimethyl-naphthalene; (15) 1,4,5-trimethyl-naphthalene; and (16) 2,7-dimethyl-quinoline.

Variation in the peak areas of identified metabolites clearly showed differences in the relative amounts of various metabolites for different individuals (Supplementary Table 1 and Figure 4A and B). The identified metabolites belonged to several distinct chemical families: aldehydes, ketones, terpenoids, acids, alcohols, benzene derivatives, furan and sulphur-containing compounds, phenols, esters, naphthalene derivatives and miscellaneous (Figure 4A).

We observed relatively consistent changes for many metabolites in samples obtained from the cancer group, with the most common pattern being decreased production (downregulation) in the cancer group samples and either increased production (upregulation) or a negligible change in the control group. For example, volatile sulphur-containing metabolites such as dimethyl disulphide, methanethiol, dimethyl trisulphide and methoxythiophene, which are generated in humans by the incomplete metabolism of methionine in the transamination pathway, were dramatically downregulated as a consequence of neoplastic cell presence (Supplementary Table 1). Thus, an overall downregulation of these metabolites may be a common feature of tumour growth.

For the control group, the largest chemical classes in the metabolomic profile were ketones, sulphur compounds, volatile phenols and terpenoids. The major metabolites of these chemical families are 4-heptanone, dimethyl disulphide, *p*-tert-butyl phenol and D-carvone, respectively. The origin of 4-heptanone is still unknown, but it is probably from an exogenous source (Deng *et al*, 2004b). It has previously been reported that 4-heptanone is produced from the *in vivo* metabolism of plasticisers in humans (Walker and Mills, 2001). Esters and higher alcohols were the chemical classes with the smallest contributions to the volatile metabolomic profile.

Ketones, volatile phenols, terpenoids and benzene derivatives were the chemical groups that contributed most to the metabolomic profile of the oncological group (Figure 4A). The most abundant metabolites that belonged to these chemical classes were 4-heptanone, *p*-tert-butyl-phenol, 2,6-dimethyl-7-octen-2-ol and 1-ethyl-3,5-diisopropyl-benzene, respectively. The average areas of these metabolites indicated that when all cancer patients were combined into a single group, patients with cancer had higher levels of 3-methyl-3-phenyl-2-propenal, 3-heptanone, *p*-cymene, 2-methoxythiophene, phenol, 4-methyl-phenol and 1,2-dihydro-1,1,6-trimethyl-naphthalene than the control group (Figure 4B). There was an increase in the amounts of all of these chemicals for all cancer patients when compared with the controls, except dimethyl disulphide, which was also a major compound that was identified to be present in the control group individuals.

The fragment ion *m/z* values of the identified urinary metabolites with the highest abundance within each fragmentation pattern, the matching percentage of the NIST library and their frequency of occurrence in cancer patients and normal controls are listed in Table 2.

Among the 82 total metabolites identified, 21 were found in all (patients and controls) of the individuals who submitted to this study. As can be seen in Table 2, of the 82 identified metabolites, only 27 were common to colorectal cancer patients, whereas in the leukaemia and lymphoma cancer patients, the total number of common metabolites was 28 and 36, respectively. 3-Heptanone was only detected in half of the lymphoma patients, whereas 4-methyl-2-heptanone was only identified in colorectal patients (84%).

One-way ANOVA with *P*_*values*_<0.05 was achieved for both groups (oncological and control) using SPSS, version 17.0. The positive rates of all 82 volatile metabolites were compared between the cancer group and the control group. Of the 82 identified metabolites, 16 were statistically significant (ANOVA, *P*<0.05) between the cancer patient and control groups, whereas the others did not show any significant differences. These volatile metabolites are summarised in Table 3.

**Figure 5:**
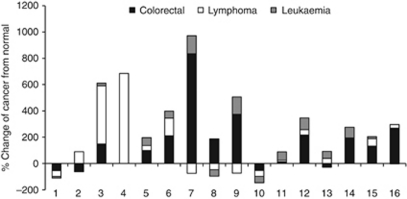
Percentage of change between cancer patients and normal subjects. Numbered bars correspond to: (1) hexanal; (2) heptanal; (3) 2-methyl-3-phenyl-2-propenal; (4) 3-heptanone; (5) 1,2,4-trimethylbenzene; (6) *p*-cymene; (7) anisole; (8) *γ*-terpinene; (9) bornylene; (10) dimethyl disulphide; (11) 2-methoxythiophene; (12) 4-methyl-phenol; (13) 1-octanol; (14) 1,2-dihydro-1,1,6-trimethyl-naphthalene; (15) 1,4,5-trimethyl-naphthalene; and (16) 2,7-dimethyl-quinoline.

Of the 16 metabolites of interest, it can be see that 3-heptanone, anisole, 2-methyl-3-phenyl-2-propenal, 2,7-dimethyl-quinoline and hexanal had the lowest *P*-values. If we looked more closely at the peak areas (Table 3), the urinary levels of 2-methyl-3-phenyl-2-propenal, 3-heptanone and anisole that were found in healthy volunteers were much lower than those found in cancer patients. On the other hand, the urinary levels of hexanal and dimethyl disulphide were much higher in healthy persons than in cancer patients (Figure 5).

Metabolomic differences between the different types of investigated cancers were also detected (Figure 5). Such differences were manifested as higher levels of 2-methyl-3-phenyl-2-propenal and 3-heptanone and lower levels of aldehydes (hexanal and heptanal) in the lymphoma cancer patients in comparison with colorectal and leukaemia patients. Furthermore, urinary levels of *p*-cymene, anisole, *γ*-terpinene, bornylene, dimethyl disulphide, 4-methyl-phenol, 1,2-dihydro-1,1,6-trimethyl-naphthalene, 1,4,5-trimethyl-naphthalene and 2,7-dimethyl-quinoline were higher in colorectal patients than in lymphoma and leukaemia patients. In addition, the increased content of sulphur-containing metabolites, namely 2-methoxythiophene and dimethyl disulphide, in leukaemia patients were statistically significant in comparison with normal controls (Figure 5).

**Figure 6:**
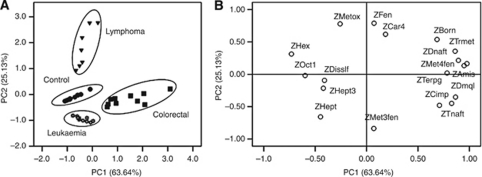
Separation of cancer patients and healthy individuals based on principal component analysis (PCA) scores scatter plot. (**A**) Loadings of variables on the PC1–PC2 plane (88.77% of total variance). (**B**) Factor scores for PCA analysis showing the similarity groups for healthy persons (control group), and cancer patients as described by variables represented in Figure 4A.

The high inter-individual variability in urinary profiles and their complexity make any attempt at visual comparison of these spectra an unproductive task. Instead, multivariate analysis allowed the finding of consistent variation patterns within the data set. To study the principal sources of variation among the results, detect intrinsic clustering and possible outliers, and distinguish cancer patients from healthy individuals, exploratory PCA was applied to the GC-qMS peak areas that were obtained for the urinary volatile metabolites of both groups. This PCA is an unsupervised projection method that is used to visualise the data set and display similarities and differences. After preliminary statistical analysis, the PCA of the data showed that the variables described in Table 3 were enough to describe subsets with similar characteristics and relate to the health status of the subjects. Figure 6A depicts the loadings of 16 variables on a plane defined by the first (PC1) and second (PC2) principal components.

Although this set of variables only explained 88.77% of the variability between the first two PCs, it was enough to deconvolute the set of cases in four subsets according to the health of the subjects and to cancer type. These results indicate a significant potential of early diagnosis of the studied cancer types using non-invasive urinary metabolomic analysis (Figure 6B).

The scores of the scatter plot demonstrate that PC2, which contained 25.13% of the total variability, split the samples into three different groups. The group located among the PC2-positive values corresponded to patients with lymphoma, whereas the second group, located among the negative values from PC1 and PC2, contained the leukaemia patients and normal controls. The third group, located among the PC1-positive and PC2-negative values, comprised the samples corresponding to the colorectal cancer subjects. These results demonstrate that the set of cases could be divided into four groups according to the clinical condition of the subjects.

The chemicals 3-heptanone (ZHept3), dimethyl disulphide (ZDisslf) and heptanal (ZHept) appear to play an important role in the healthy individuals group (control) and leukaemia patients. Colorectal cancer patients appear to be better characterised by 1,4,5-trimethyl-naphthalene (ZTnaft), 2,7-dimethyl-quinoline (ZDmql) and 2-methyl-3-phenyl-2-propenal (ZMet3fen). The chemicals 1-octanol (ZOct1), hexanal (ZHex) and 2-methoxythiophene (ZMetox) correlated well with the lymphoma subjects.

## Conclusions

In this study, dHS-SPME/GC-qMS-based metabolomics was applied to investigate urinary cancer volatile metabolites as a useful tool to evaluate volatile metabolites for the early diagnosis of cancer. We found that dHS-SPME combined with GC-qMS was a simple, rapid, sensitive and solvent-free method that is highly suitable for this purpose. The potential advantages of urine analysis over other conventional medical tests include its non-invasive nature, low cost and safety. To achieve the highest recovery rate, the isolation procedure was optimised via the selection of an appropriate fibre, extraction temperature and extraction time using univariate optimisation design. A CAR/PDMS fibre was found to be more sensitive for volatile metabolites than other coating phases. In all, 82 volatile metabolites were detected and identified in the control and oncological groups belonging to distinct chemical families, including aldehydes, ketones, terpenoids, acids, alcohols, benzene derivatives, furans and sulphur compounds, phenols, esters and naphthalene derivatives. Some of the metabolites were of endogenous origin and generated in the human body during normal metabolic processes, whereas some were inhaled with subsequent storage and later exhalation/excretion.

For the control group, the chemical families with the higher contributions to the urinary metabolomic profile were ketones and sulphur compounds. The major metabolites of these chemical families were 4-heptanone and methanethiol. Benzene derivatives, terpenes and volatile phenols were the chemical classes that exhibited higher contributions to the metabolomic patterns that were identified in the oncological group. Different VOM profiles for healthy people and cancer patients could be recognised by multivariate analysis, and significant diagnostic compounds could be established. Between the cancer group (*n*=33) and the control group (*n*=21), positive rates for 16 of the 82 detected compounds were found to be different with statistical significance (*P*<0.05, LSD test). There were definitive metabolomic differences between cancer patients and healthy individuals and between different types of cancer. The identification of volatile biomarkers in urine for disease diagnosis is an area of great promise, but it is based on limited previous human research. The data in this paper are consistent with the hypothesis that diagnostically useful volatile compounds are produced in patients with cancer and secreted into the urine, thus providing support for this diagnostic approach. The ability to easily collect and store urine samples will be a major advantage of this approach.

## Figures and Tables

**Table 1:** The characteristics of subjects

	**Patients**			
	**Colon**	**Leukaemia**	**Lymphoma**	**Normal controls**
Number	11	14	7	21
Mean age (s.d.)	62.0 (11.7)	50.1 (12.4)	42.0 (19.1)	44.1 (10.3)
Gender (male/female)	8/3	6/8	6/1	18/3
Current smokers	0	0	2	3
Ex-smokers	0	0	1	0

Abbreviation: s.d.= standard deviation.

**Table 2:** Identification mode, fragment ion *m/z* with the highest abundance match percentage to the NIST library and the frequency of occurrence of the identified metabolites in patients and normal controls

				**Frequency of occurrence (%)**			
**Metabolites**	**ID^a^**	***m*/*z***	**Match per cent**	**Colorectal**	**Leukaemia**	**Lymphoma**	**Control**
Methanethiol^b^	MS	47	80	100.0^c^	100.0	100.0	100.0
Furan^b^	St, MS	68	91	100.0	100.0	100.0	100.0
Acetone^b^	St, MS	43	80	100.0	100.0	100.0	100.0
2-Methylfuran^b^	MS	82	87	100.0	100.0	100.0	100.0
Ethyl acetate	St, MS	43	85	33.3	71.4	71.4	71.4
2-Butanone^b^	St, MS	43	82	100.0	100.0	100.0	100.0
2-Methyl-butanal	MS	57	87	33.3	42.9	57.1	71.4
3-Methyl-butanal	St, MS	44	81	33.3	7.1	14.3	28.6
2,5-Dimethyl-furan	MS	96	85	91.7	100.0	100.0	100.0
2-Pentanone^b^	St, MS	43	86	100.0	100.0	100.0	100.0
Methyl isobutyl ketone	St, MS	43	93	41.7	71.4	71.4	100.0
Toluene	St, MS	91	86	91.7	100.0	85.7	100.0
1-(2-furanyl)ethanone^b^	St, MS	95	80	100.0	100.0	100.0	100.0
Dimethyl disulphide^b^	St, MS	94	97	100.0	100.0	100.0	100.0
3-Hexanone^b^	St, MS	43	84	100.0	100.0	100.0	100.0
Hexanal^b^	St, MS	44	90	100.0	100.0	100.0	100.0
Geraniol oxide	MS	139	90	41.7	57.1	57.1	90.5
4-Heptanone^b^	St, MS	71	91	100.0	100.0	100.0	100.0
*p*-Xylene	MS	91	88	16.7	21.4	42.9	NF
3-Heptanone	St, MS	57	89	NF	NF	57.1	95.2
*α*-Terpinene	St, MS	121	95	75.0	85.7	71.4	52.4
1,4-Cineol	St, MS	111	87	58.3	78.6	85.7	61.9
Limonene	St, MS	68	84	33.3	57.1	57.1	28.6
2-Heptanone	St, MS	43	83	91.7	92.9	85.7	100.0
Heptanal	St, MS	44	80	91.7	NF	14.3	95.2
4-Methyl-2-heptanone	MS	43	80	84.0	NF	NF	19.4
*γ*-Terpinene	St, MS	93	89	58.3	92.9	100.0	57.1
*m*-Cymene^b^	MS	119	97	100.0	100.0	100.0	100.0
2,2,6-Trimethyl-cyclohexanone	MS	82	88	41.7	71.4	42.9	76.2
2-Methoxythiophene	MS	114	89	100.0	92.9	100.0	100.0
1,2,4-Trimethylbenzene	MS	105	90	83.3	85.7	100.0	95.2
Dimethyl trisulphide	St, MS	126	91	83.3	100.0	100.0	100.0
2-Methyl-5-(methylthio)furan	MS	128	91	91.7	92.9	85.7	100.0
Nonanal	St, MS	57	80	83.3	100.0	85.7	100.0
1,2,3,4-Tetrahydro-1,5,7-trimethyl-naphthalene	MS	159	90	66.7	71.4	85.7	81.0
*p*-Cymene	MS	132	97	91.7	100.0	100.0	100.0
Linalyl oxide	MS	59	82	83.3	92.9	100.0	90.5
Dihydrolinalool	MS	73	84	16.7	28.6	57.1	76.2
Acetic acid^b^	St, MS	43	90	100.0	100.0	100.0	100.0
Furfural	St, MS	96	91	100.0	92.9	100.0	100.0
2,6-Dimethyl-7-octen-2-ol	St, MS	59	90	91.7	100.0	100.0	100.0
1,2,3,4-Tetramethyl-benzene	MS	119	80	50.0	28.6	57.1	66.7
Decanal	St, MS	57	86	58.3	85.7	71.4	100.0
Bornylene	MS	93	96	83.3	85.7	85.7	71.4
Vitispirane I^b^	MS	192	82	100.0	100.0	100.0	100.0
Vitispirane II^b^	MS	192	85	100.0	100.0	100.0	100.0
1,2,3,4-Tetrahydro-1,1,6-trimethyl-naphthalene	MS	159	92	91.7	92.9	100.0	100.0
1-Octanol	St, MS	56	90	91.7	85.7	71.4	100.0
Menthol	MS	71	94	83.3	85.7	57.1	71.4
2-Furanmethanol	St, MS	98	80	83.3	78.6	57.1	85.7
2-Methyl butanoic acid	St, MS	74	83	91.7	92.9	100.0	81.0
Anisole	MS	134	95	83.3	85.7	71.4	71.4
(+)-4-Carene	MS	93	80	58.3	64.3	28.6	71.4
2-Methyl-3-phenyl-2-propenal	St, MS	145	80	91.7	85.7	100.0	100.0
4-(1-Methylethyl)-1-cyclohexene-4-carboxaldehyde	MS	109	85	41.7	42.9	57.1	19.0
3-Carvomenthenone	MS	82	80	33.3	42.9	28.6	33.3
D-Carvone	MS	82	80	66.7	57.1	57.1	52.4
1,2-Dihydro-1,1,6-trimethyl-naphthalene^b^	St, MS	157	97	100.0	100.0	100.0	100.0
1-(4-Methylphenyl)ethanone	MS	119	94	33.3	28.6	14.3	14.3
1-Decanol	MS	56	88	25.0	28.6	14.3	0.0
4-(1-methylethyl)-benzaldehyde	MS	133	84	58.3	14.3	57.1	9.5
3,4-Dimethyl-benzaldehyde^b^	MS	133	95	100.0	100.0	100.0	100.0
*β*-Damascenone^b^	St, MS	69	86	100.0	100.0	100.0	100.0
*p*-Cymene-8-ol	MS	43	90	66.7	85.7	100.0	100.0
2-Methoxy-phenol	St, MS	109	80	91.7	92.9	100.0	66.7
2,7-Dimethyl-quinoline	St, MS	157	85	83.3	85.7	85.7	85.7
Hexanoic acid	St, MS	60	90	50.0	50.0	71.4	90.5
1-Dodecanol	MS	55	89	8.3	7.1	14.3	NF
2,6-Dimethyl-naphthalene	MS	156	98	100.0	92.9	71.4	76.2
1-Ethyl-3,5-diisopropyl-benzene	MS	175	88	100.0	100.0	100.0	NF
Phenol^b^	St, MS	94	91	100.0	100.0	100.0	100.0
Eugenol	St, MS	164	92	33.3	28.6	NF	NF
Octanoic acid	St, MS	60	83	91.7	85.7	85.7	95.2
4-Methyl-phenol	St, MS	107	90	100.0	100.0	85.7	95.2
Hexadecanal	MS	57	96	33.3	NF	28.6	NF
1,4,5-Trimethyl-naphthalene	MS	155	86	66.7	78.6	71.4	57.1
2-Methoxy-4-vinylphenol	St, MS	135	80	33.3	42.9	28.6	52.4
Decanoic acid	St, MS	60	80	66.7	71.4	71.4	100.0
*p*-Tert-butyl-phenol^b^	MS	135	96	100.0	100.0	100.0	100.0
2,4-Bis(1,1-dimethylethyl)-phenol	MS	191	95	100.0	92.9	85.7	100.0
Benzenecarboxilic acid	St, MS	105	90	58.3	64.3	71.4	85.7
Indole	MS	117	88	100.0	100.0	100.0	100.0

Abbreviation: NF= not found.

a Metabolite identification using standard compound (st) or mass spectra of the NIST library search (MS).

b Metabolites identified in all 54 studied subject.

c Means that the metabolite was identified in all subjects of the corresponding group.

**Table 3:** Potential volatile marker metabolites found in the urinary volatile composition of the four groups by total significance of *one-way* ANOVA and LSD *post-hoc* test for multiple comparisons

	**Mean^a^ values of peak areas (*n*=3)**							**One-way significance**		
**Metabolites**	**Colon (A)**	**%Change^b^**	**Lymphoma (B)**	**%Change**	**Leukaemia (D)**	**%Change**	**Control (E)**	**F**	***P*-value^c^**	**LSD (multiple comparison test)**
Hexanal	101 409	−54.6	125 271	−44.0	195 841	−12.4	223 543	4.531	0.002	A–E (*P*=0.016)
Heptanal	17 866	−62.3	89 807	89.6	NF	NF	47 370	4.084	0.024	A–E (*P*=0.045)
2-Methyl-3-phenyl-2-propenal	299 264	148.3	653 443	442.2	144 933	20.2	120 528	5.515	0.001	A–B (*P*=0.012)
										B–D (*P*<0.001)
										B–E (*P*<0.001)
3-Heptanone	NF	NF	566 713	684.9	NF	NF	72 205	16.490	<0.001	B–E (*P*=0.002)
1,2,4-Trimethylbenzene	82 898	99.2	57 260	37.6	66 229	59.1	41 623	3.124	0.032	A–E (*P*=0.006)
*p*-Cymene	1743 047	210.2	1 320 992	135.1	858 328	52.8	561 906	2.741	0.050	A–E (*P*=0.009)
Anisole	568 939	834.5	14 948	−75.4	144 401	137.2	60 881	23.088	<0.001	A–D (*P*<0.001)
										A–E ( *P*<0.001)
*γ*-Terpinene	499 986	187.2	91 305	−47.5	88 595	−49.1	174 078	4.041	0.012	A–D (*P*=0.004)
										A–E (*P*=0.022)
Bornylene	73 702	374.3	3998	−74.3	35 962	131.4	15 540	3.131	0.047	A–D (*P*=0.026)
										A–E (*P*=0.009)
Dimethyl disulphide	3 246 008	−52.9	3 789 218	−45.0	3 496 595	−49.3	6 892 194	4.667	0.005	A–E (*P*=0.006)
										D–E (*P*=0.008)
2-Methoxythiophene	282 352	14.1	2 767 00	11.9	401 862	62.4	247 376	5.211	0.003	D–E (*P*=0.005)
4-Methyl-phenol	3 730 154	216.5	1 660 457	40.9	2 232 899	89.4	1178 693	3.776	0.014	A–E (*P*=0.006)
1-Octanol	64 089	−28.8	124 984	38.9	136 895	52.2	89 968	3.562	0.019	A–D (*P*=0.002)
										D–E (*P*=0.021)
1,2-Dihydro-1,1, 6-trimethyl-naphthalene	2 092 233	189.0	760 528	5.1	1 316 780	81.9	723 911	3.571	0.018	A–E (*P*=0.002)
1,4,5-Trimethyl-naphthalene	75 610	131.7	51 364	57.4	37 398	14.6	32 634	4.136	0.011	A–D (*P*=0.006)
										A–E (*P*=0.002)
2,7-Dimethyl-quinoline	326 614	268.1	113 090	27.5	85 692	−3.4	88 718	5.697	0.002	A–D (*P*=0.002)
										A–E (*P*=0.001)

Abbreviations: ANOVA= analysis of variance; LSD= least significant difference; NF= not found; r.s.d.= relative standard deviation.

a Average value from three replicates; r.s.d. lower than 20%.

b Percentage change of cancer from normal, calculated from the arithmetic mean values of each group. Positive and negative percentages indicate higher levels of metabolites in cancer patients and healthy subjects, respectively.

c Statistical *P-*value calculated using the LSD test (significance at *P*<0.05).

## References

[bib1] Barrett JC, Anderson M (1993) Molecular mechanisms of carcinogenesis in humans and rodents. Mol Carcinogen 7: 1–13 10.1002/mc.2940070102 8094618

[bib2] Belda-Iniesta C, de Castro Carpeno J, Carrasco JA, Moreno V, Casado Saenz E, Feliu J, Sereno M, Garcia Rio F, Barriuso J, Gonzalez Baron M (2007) New screening method for lung cancer by detecting volatile organic compounds in breath. Clin Transl Oncol 9: 364–368 17594950 10.1007/s12094-007-0068-6

[bib3] Carrola J, Rocha CM, Barros AS, Gil AM, Goodfellow BJ, Carreira IM, Bernardo J, Gomes A, Sousa V, Carvalho L, Duarte IF (2011) Metabolic signatures of lung cancer in biofluids: NMR-based metabonomics of urine. J Proteome Res 10: 221–230 21058631 10.1021/pr100899x

[bib4] Cassiday LT (2006) Weighing ribosomes with MS. Anal Chem 78: 7926 17186634

[bib5] Chen J, Wang W, Lv S, Yin P, Zhao X, Lu X, Zhang F, Xu G (2009) Metabonomics study of liver cancer based on ultra performance liquid chromatography coupled to mass spectrometry with HILIC and RPLC separations. Anal Chim Acta 650: 3–9 19720165 10.1016/j.aca.2009.03.039

[bib6] Chen X, Xu F, Wang Y, Pan Y, Lu D, Wang P, Ying K, Chen E, Zhang W (2007) A study of the volatile organic compounds exhaled by lung cancer cells *in vitro* for breath diagnosis. Cancer 110: 835–844 17599760 10.1002/cncr.22844

[bib7] Chikkaveeraiah BV, Bhirde A, Malhotra R, Patel V, Gutkind JS, Rusling JF (2009) Single-wall carbon nanotube forest arrays for immunoelectrochemical measurement of four protein biomarkers for prostate cancer. Anal Chem 81: 9129–9134 19775154 10.1021/ac9018022 PMC2901508

[bib8] Deng C, Li N, Zhang X (2004a) Development of headspace solid-phase microextraction with on-fiber derivatization for determination of hexanal and heptanal in human blood. J Chromatogr B 813: 47–52 10.1016/j.jchromb.2004.09.007 15556514

[bib9] Deng C, Zhang X, Li N (2004b) Investigation of volatile biomarkers in lung cancer blood using solid-phase microextraction and capillary gas chromatography-mass spectrometry. J Chromatogr B 808: 269–277 10.1016/j.jchromb.2004.05.015 15261821

[bib10] Gaspar EM, Lopes JF (2009) Simple gas chromatographic method for furfural analysis. J Chromatogr A 1216: 2762–2767 18976770 10.1016/j.chroma.2008.10.049

[bib11] Giardina M, Olesik SV (2003) Application of low-temperature glassy carbon-coated macrofibers for solid-phase microextraction analysis of simulated breath volatiles. Anal Chem 75: 1604–1614 12705592 10.1021/ac025984k

[bib12] Gramolini AO, Kislinger T, Alikhani-Koopaei R, Fong V, Thompson NJ, Isserlin R, Sharma P, Oudit GY, Trivieri MG, Fagan A, Kannan A, Higgins DG, Huedig H, Hess G, Arab S, Seidman JG, Seidman CE, Frey B, Perry M, Backx PH, Liu PP, MacLennan DH, Emili A (2008) Comparative proteomics profiling of a phospholamban mutant mouse model of dilated cardiomyopathy reveals progressive intracellular stress responses. Mol Cell Proteomics 7: 519–533 18056057 10.1074/mcp.M700245-MCP200

[bib13] Greenberg AK, Lee MS (2007) Biomarkers for lung cancer: clinical uses. Curr Opin Pulm Med 13: 249–255 17534168 10.1097/MCP.0b013e32819f8f06

[bib14] Henneges C, Bullinger D, Fux R, Friese N, Seeger H, Neubauer H, Laufer S, Gleiter CH, Schwab M, Zell A, Kammerer B (2009) Prediction of breast cancer by profiling of urinary RNA metabolites using support vector machine-based feature selection. BMC Cancer 9: 104 19344524 10.1186/1471-2407-9-104 PMC2680413

[bib15] Infante PF, Schuman LD, Dement J, Huff J (1994) Fibrous glass and cancer. Am J Ind Med 26: 559–584 7810554 10.1002/ajim.4700260413

[bib16] Jemal A, Siegel R, Xu J, Ward E (2010) Cancer statistics, 2010. CA Cancer J Clin 60: 277–300 20610543 10.3322/caac.20073

[bib17] Jentzmik F, Stephan C, Miller K, Schrader M, Erbersdobler A, Kristiansen G, Lein M, Jung K (2010) Sarcosine in urine after digital rectal examination fails as a marker in prostate cancer detection and identification of aggressive tumours. Eur Urol 58: 12–18; discussion 20–1 20117878 10.1016/j.eururo.2010.01.035

[bib18] Jiang Y, Ma Y (2009) A fast capillary electrophoresis method for separation and quantification of modified nucleosides in urinary samples. Anal Chem 81: 6474–6480 19552424 10.1021/ac901216n

[bib19] Lauridsen M, Hansen SH, Jaroszewski JW, Cornett C (2007) Human urine as test material in ^1^H NMR-based metabonomics: recommendations for sample preparation and storage. Anal Chem 79: 1181–1186 17263352 10.1021/ac061354x

[bib20] Ma YL, Qin HL, Liu WJ, Peng JY, Huang L, Zhao XP, Cheng YY (2009) Ultra-high performance liquid chromatography-mass spectrometry for the metabolomic analysis of urine in colorectal cancer. Dig Dis Sci 54: 2655–2662 19117128 10.1007/s10620-008-0665-4

[bib21] Matsumura K, Opiekun M, Oka H, Vachani A, Albelda SM, Yamazaki K, Beauchamp GK (2010) Urinary volatile compounds as biomarkers for lung cancer: a proof of principle study using odor signatures in mouse models of lung cancer. PLoS One 5: e8819 20111698 10.1371/journal.pone.0008819 PMC2811722

[bib22] Mazzone PJ (2008) Analysis of volatile organic compounds in the exhaled breath for the diagnosis of lung cancer. J Thorac Oncol 3: 774–780 18594325 10.1097/JTO.0b013e31817c7439

[bib23] McCulloch M, Jezierski T, Broffman M, Hubbard A, Turner K, Janecki T (2006) Diagnostic accuracy of canine scent detection in early- and late-stage lung and breast cancers. Integr Cancer Ther 5: 30–39 16484712 10.1177/1534735405285096

[bib24] Minamoto T, Mai M, Ronai Z (2000) K-ras mutation: early detection in molecular diagnosis and risk assessment of colorectal, pancreas, and lung cancers-a review. Cancer Detect Prev 24: 1–12 10757118

[bib25] Musteata FM, Pawliszyn J (2007) Bioanalytical applications of solid-phase microextraction. Trends Anal Chem 26: 36–45

[bib26] Oliveira PA, Colaco A, Chaves R, Guedes-Pinto H, De-La-Cruz PL, Lopes C (2007) Chemical carcinogenesis. An Acad Bras Cienc 79: 593–616 18066431 10.1590/s0001-37652007000400004

[bib27] Ouyang G, Chen Y, Setkova L, Pawliszyn J (2005) Calibration of solid-phase micro-extraction for quantitative analysis by gas chromatography. J Chromatogr A 1097: 9–16 16298180 10.1016/j.chroma.2005.08.017

[bib28] Phillips M, Cataneo RN, Ditkoff BA, Fisher P, Greenberg J, Gunawardena R, Kwon CS, Rahbari-Oskoui F, Wong C (2003) Volatile markers of breast cancer in the breath. Breast J 9: 184–191 12752626 10.1046/j.1524-4741.2003.09309.x

[bib29] Phillips M, Cataneo RN, Ditkoff BA, Fisher P, Greenberg J, Gunawardena R, Kwon CS, Tietje O, Wong C (2006) Prediction of breast cancer using volatile biomarkers in the breath. Breast Cancer Res Treat 99: 19–21 16502014 10.1007/s10549-006-9176-1

[bib30] Prado C, Marin P, Periago JF (2003) Application of solid-phase microextraction and gas chromatography-mass spectrometry to the determination of volatile organic compounds in end-exhaled breath samples. J Chromatogr A 1011: 125–134 14518769 10.1016/s0021-9673(03)01103-8

[bib31] Qiu Y, Cai G, Su M, Chen T, Liu Y, Xu Y, Ni Y, Zhao A, Cai S, Xu LX, Jia W (2010) Urinary metabonomic study on colorectal cancer. J Proteome Res 9: 1627–1634 20121166 10.1021/pr901081y

[bib32] Song G, Qin T, Liu H, Xu GB, Pan YY, Xiong FX, Gu KS, Sun GP, Chen ZD (2010) Quantitative breath analysis of volatile organic compounds of lung cancer patients. Lung Cancer 67: 227–231 19409642 10.1016/j.lungcan.2009.03.029

[bib33] Sreekumar A, Poisson LM, Rajendiran TM, Khan AP, Cao Q, Yu J, Laxman B, Mehra R, Lonigro RJ, Li Y, Nyati MK, Ahsan A, Kalyana-Sundaram S, Han B, Cao X, Byun J, Omenn GS, Ghosh D, Pennathur S, Alexander DC, Berger A, Shuster JR, Wei JT, Varambally S, Beecher C, Chinnaiyan AM (2009) Metabolomic profiles delineate potential role for sarcosine in prostate cancer progression. Nature 457: 910–914 19212411 10.1038/nature07762 PMC2724746

[bib34] Ullah MF, Aatif M (2009) The footprints of cancer development: cancer biomarkers. Cancer Treat Rev 35: 193–200 19062197 10.1016/j.ctrv.2008.10.004

[bib35] Walker V, Mills GA (2001) Urine 4-heptanone: a beta-oxidation product of 2-ethylhexanoic acid from plasticisers. Clin Chim Acta 306: 51–61 11282094 10.1016/s0009-8981(01)00390-4

[bib36] Weisburger JH, Williams GM (2000) The distinction between genotoxic and epigenetic carcinogens and implication for cancer risk. Toxicol Sci 57: 4–5 10966505 10.1093/toxsci/57.1.4

[bib37] Xue R, Dong L, Zhang S, Deng C, Liu T, Wang J, Shen X (2008) Investigation of volatile biomarkers in liver cancer blood using solid-phase microextraction and gas chromatography/mass spectrometry. Rapid Commun Mass Spectrom 22: 1181–1186 18350562 10.1002/rcm.3466

[bib38] Yu Q, Huang R, Li L, Lin L, Hang W, He J, Huang B (2009) Applicability of standardless semiquantitative analysis of solids by high-irradiance laser ionization orthogonal time-of-fight mass spectrometry. Anal Chem 81: 4343–4348 19408955 10.1021/ac900141z

[bib39] Yung TCH (2010) Is that lung cancer I smell in your breath? Univ Toronto Med J 87: 122–123

