# Supplementary material for: Investigation of urinary volatile organic metabolites as potential cancer biomarkers by solid-phase microextraction in combination with gas chromatography-mass spectrometry
Source: Br J Cancer. 2011 Nov 15;105(12):1894–904. doi: 10.1038/bjc.2011.437 (PMC3251876; doi:10.1038/bjc.2011.437)
Supplement: Supplementary Table 1 [file bjc2011437x1.doc]

**Supplementary table 1 GC-qMS peak areas of the volatile metabolites identified in cancer patients and healthy subjects**

| **RT (min)^a^** | **Chemical classes / Metabolites** | **Colorectal** | | | | **Lymphoma** | | | | **Leukemia** | | | | **Control** | | | |
| --- | --- | --- | --- | --- | --- | --- | --- | --- | --- | --- | --- | --- | --- | --- | --- | --- | --- |
|  | **Aldehydes** | **Min** | **Max** | **Median** | **RSD %** | **Min** | **Max** | **Median** | **RSD %** | **Min** | **Max** | **Median** | **RSD %** | **Min** | **Max** | **Median** | **RSD %** |
| 3.044 | 2-Methyl-butanal | 20854 | 84484 | 49599 | 9.7 | 9369 | 45623 | 19422 | 7.7 | 22882 | 53851 | 39037 | 7.2 | 16213 | 158349 | 37140 | 7.3 |
| 3.106 | 3-Methyl-butanal | 27910 | 44074 | 35915 | 20.4 | NF- | NF | NF |  | NF | NF | NF |  | 22217 | 280998 | 47162 | 6.5 |
| 6.308 | Hexanal | 54705 | 169787 | 94781 | 9.8 | 65190 | 207610 | 93590 | 9.2 | 51835 | 493187 | 146752 | 10.3 | 86008 | 542292 | 211735 | 14.7 |
| 10.753 | Heptanal | 13620 | 22113 | 17866 | 19.9 | NF | NF | NF | - | NF | NF | NF |  | 27251 | 81295 | 42611 | 18.6 |
| 20.917 | Nonanal | 39088 | 150825 | 82375 | 12.5 | 70098 | 235397 | 141525 | 13.3 | 25891 | 292502 | 90579 | 10.1 | 42384 | 176708 | 103981 | 21.2 |
| 26.341 | Decanal | 53363 | 883535 | 501171 | 4.5 | 110522 | 1278447 | 674481 | 9.4 | 33414 | 1588855 | 918289 | 9.9 | 263715 | 881988 | 459705 | 15.0 |
| 35.983 | 2-Methyl-3-phenyl-2-propenal | 23859 | 1710466 | 109546 | 8.1 | 17674 | 1765441 | 610224 | 8.3 | 40581 | 373819 | 119340 | 10.7 | 28108 | 525689 | 76906 | 14.5 |
| 39.426 | 4-(1-methylethyl)-benzaldehyde | 20919 | 828418 | 49958 | 15.2 | 18182 | 123639 | 38409 | 10.0 | 67283 | 90773 | 79028 | 16.4 | 87882 | 104355 | 96118 | 8.4 |
| 40.757 | 3,4-Dimethyl-benzaldehyde | 93479 | 546240 | 146226 | 8.9 | 124028 | 3013191 | 163614 | 9.9 | 71406 | 712789 | 258054 | 10.1 | 106262 | 1188757 | 522090 | 20.8 |
| 54.691 | Hexadecanal | 28089 | 170447 | 78763 | 10.5 | 16524 | 193229 | 104876 | 7.5 | NF | NF | NF |  | NF | NF | NF | - |
|  | **Mean** | **37588** | **461039** | **116620** |  | **53948** | **857822** | **230768** |  | **44756** | **515111** | **235868** |  | **75560** | **437825** | **177494** |  |
|  | **Ketones** |  |  |  |  |  |  |  |  |  |  |  |  |  |  |  |  |
| 2.127 | Acetone | 330181 | 5198420 | 1434954 | 10.1 | 397260 | 2096667 | 627453 | 7.3 | 371665 | 6024489 | 861229 | 10.1 | 395130 | 1617827 | 1092778 | 13.9 |
| 2.983 | 2-Butanone | 93206 | 2323788 | 556596 | 9.4 | 72375 | 1715067 | 637309 | 10.2 | 131220 | 2761893 | 757743 | 10.2 | 162781 | 3467578 | 1212146 | 13.3 |
| 3.831 | 2-Pentanone | 94902 | 6504760 | 1714922 | 9.3 | 122468 | 8301725 | 1501719 | 9.3 | 420067 | 15226577 | 2042483 | 8.1 | 516424 | 11842822 | 2717415 | 14.8 |
| 4.722 | Methyl isobutyl ketone | 79260 | 111251 | 85533 | 5.6 | 22056 | 84778 | 57507 | 5.6 | 32144 | 147715 | 84819 | 6.5 | 23242 | 220246 | 74908 | 15.7 |
| 5.504 | 1-(2-furanyl)ethanone | 85553 | 5555126 | 259990 | 11.5 | 85316 | 411951 | 297903 | 8.4 | 90951 | 2154907 | 363700 | 10.6 | 100054 | 999952 | 365525 | 15.2 |
| 6.009 | 3-Hexanone | 23071 | 161679 | 107167 | 7.3 | 34957 | 388808 | 91807 | 7.6 | 35973 | 343751 | 136790 | 10.9 | 51666 | 353137 | 139586 | 15.1 |
| 7.886 | 4-Heptanone | 613833 | 4661510 | 3340755 | 8.0 | 2105743 | 24172886 | 3456320 | 7.8 | 2014548 | 14885468 | 3840998 | 9.8 | 1424652 | 8682932 | 6350433 | 13.9 |
| 9.135 | 3-Heptanone | NF | NF | NF | - | 90997 | 1376602 | 399627 | 10.0 | NF | NF | NF | - | 40689 | 177942 | 60948 | 16.0 |
| 10.403 | 2-Heptanone | 57394 | 423694 | 108860 | 13.1 | 99447 | 621477 | 163543 | 12.7 | 80486 | 564548 | 125232 | 9.2 | 36478 | 279197 | 130412 | 16.5 |
| 11.364 | 4-Methyl-2-heptanone | NF | NF | NF | - | NF | NF | NF | - | NF | NF | NF | - | 35618 | 73401 | 49861 | 3.5 |
| 14.159 | 3-Octanone | 39296 | 66387 | 52842 | 4.0 | NF | NF | NF | - | NF | NF | NF | - | NF | NF | NF | - |
| 16.387 | 2,2,6-trimethyl-ciclohexanone | 11966 | 105469 | 38203 | 4.9 | 12410 | 29019 | 13278 | 4.9 | 12252 | 132925 | 28607 | 10.8 | 11069 | 78498 | 28499 | 14.1 |
|  | **Mean** | **142866** | **2511208** | **769982** |  | **304303** | **3919898** | **724646** |  | **354367** | **4693586** | **915733** |  | **254345** | **2526685** | **1111137** |  |
|  | **Benzene derivatives** |  |  |  |  |  |  |  |  |  |  |  |  |  |  |  |  |
| 5.081 | Toluene | 45329 | 538374 | 134075 | 8.9 | 162219 | 266133 | 210523 | 6.0 | 52835 | 357856 | 158154 | 10.9 | 106445 | 450104 | 187468 | 16.5 |
| 8.787 | *p*-xylene | 12434 | 106760 | 59597 | 12.8 | 37412 | 208635 | 73470 | 7.3 | 11007 | 64582 | 41857 | 9.7 | NF | NF | NF | - |
| 13.973 | *m*-cymene | 170626 | 17559498 | 978175 | 7.2 | 296513 | 8254986 | 1490101 | 12.3 | 328985 | 3582577 | 782889 | 10.6 | 223035 | 2326511 | 1047252 | 20.7 |
| 17.395 | 1,2,4-Trimethylbenzene | 23784 | 125701 | 89827 | 9.7 | 24962 | 121152 | 37173 | 11.3 | 31990 | 125386 | 49665 | 10.4 | 13208 | 158730 | 38999 | 15.7 |
| 22.985 | *p*-cymene | 115273 | 8411353 | 641188 | 10.7 | 175170 | 3354339 | 1262360 | 7.3 | 138244 | 2502461 | 496392 | 7.8 | 101769 | 1688696 | 450971 | 23.8 |
| 25.523 | 1,2,3,4-Tetramethyl-benzene | 29300 | 827331 | 207114 | 7.0 | 130317 | 420954 | 167027 | 12.6 | 24086 | 196727 | 112884 | 5.0 | 21684 | 390872 | 50326 | 20.0 |
| 35.119 | Anisole | 559561 | 578316 | 568939 | 19.4 | NF | NF | NF | - | 13870 | 384044 | 89845 | 2.3 | 22883 | 135668 | 24093 | 5.5 |
| 39.229 | 1-(4-methylphenyl)ethanone | 26397 | 151346 | 44939 | 9.2 | NF | NF | NF | - | 42774 | 57509 | 51757 | 16.0 | 42721 | 53473 | 43490 | 1.5 |
| 42.974 | *p*-Cymen-8-ol | 14059 | 598989 | 208572 | 9.5 | 41324 | 557693 | 106810 | 10.7 | 22838 | 445562 | 69782 | 8.6 | 13286 | 199732 | 77908 | 19.8 |
| 49.031 | 1-ethyl-3,5-diisopropyl-benzene | 70717 | 1048970 | 561383 | 9.0 | 113535 | 297448 | 129968 | 3.9 | 164700 | 755821 | 477755 | 8.3 | 19503 | 1122330 | 212451 | 16.8 |
|  | **Mean** | **106748** | **2994664** | **349381** |  | **122681** | **1685167** | **434679** |  | **83133** | **847252** | **233098** |  | **62726** | **725124** | **236995** |  |
|  | **Terpenoids** |  |  |  |  |  |  |  |  |  |  |  |  |  |  |  |  |
| 9.464 | α-Terpinene | 10680 | 1384065 | 40577 | 7.1 | 14817 | 138093 | 91260 | 7.3 | 12786 | 357982 | 57962 | 7.8 | 16052 | 615633 | 86466 | 13.6 |
| 9.885 | 1,4-Cineol | 11099 | 262951 | 40077 | 14.8 | 9945 | 59133 | 32075 | 13.0 | 10127 | 239140 | 35206 | 9.3 | 10422 | 1118804 | 46092 | 11.6 |
| 10.362 | Limonene | 22933 | 32826 | 23263 | 14.9 | 11509 | 30276 | 18861 | 13.5 | 8236 | 143354 | 22135 | 11.7 | 14645 | 97871 | 28795 | 8.8 |
| 13.422 | g-Terpinene | 13819 | 1955800 | 87541 | 12.1 | 11463 | 132746 | 110506 | 11.4 | 13077 | 274215 | 49396 | 10.2 | 18208 | 765721 | 76760 | 12.2 |
| 23.497 | Linalool oxide | 17119 | 4236480 | 250357 | 10.2 | 16528 | 238838 | 54217 | 13.1 | 46201 | 1468312 | 129801 | 8.3 | 12486 | 1295580 | 223411 | 11.5 |
| 23.561 | Dihydrolinalool | 86345 | 267846 | 177095 | 10.4 | 38988 | 845369 | 315038 | 11.1 | 20290 | 320643 | 136044 | 10.2 | 19638 | 2163662 | 106462 | 19.6 |
| 25.361 | 2,6-dimethyl-7-octen-2-ol | 173070 | 6946477 | 781505 | 10.3 | 178910 | 4697866 | 900617 | 8.5 | 63312 | 10881605 | 297413 | 10.6 | 71613 | 4896894 | 640225 | 15.6 |
| 27.102 | Bornylene | 36471 | 106634 | 75852 | 9.5 | NF | NF | NF | - | 17834 | 78832 | 34876 | 11.9 | 11842 | 22528 | 12249 | 10.5 |
| 27.127 | Vitispirane I | 43076 | 3756593 | 348690 | 7.4 | 66299 | 1446767 | 116763 | 7.6 | 75172 | 7965676 | 311693 | 10.6 | 58601 | 1321230 | 309251 | 16.5 |
| 27.273 | Vitispirane II | 22083 | 1669068 | 181354 | 7.1 | 31224 | 422553 | 50661 | 9.7 | 29315 | 1939210 | 122542 | 7.8 | 27006 | 784413 | 101165 | 18.2 |
| 33.297 | Menthol | 14893 | 1379031 | 448485 | 15.2 | 79513 | 232166 | 113381 | 8.0 | 52770 | 2372234 | 223680 | 10.6 | 49421 | 352426 | 147851 | 10.4 |
| 35.409 | (+)-4-Carene | 20757 | 121110 | 57424 | 10.4 | 21524 | 35860 | 28692 | 5.9 | 21841 | 243376 | 46077 | 7.8 | 13638 | 106263 | 31088 | 13.7 |
| 36.821 | 3-Carvomentenone | 28017 | 159188 | 70862 | 7.8 | 30432 | 64338 | 47385 | 2.8 | 23198 | 942202 | 187939 | 6.3 | 23233 | 84096 | 57420 | 8.3 |
| 37.209 | D-Carvone | 128059 | 3177038 | 933926 | 11.8 | 30406 | 7271883 | 1184071 | 3.3 | 209023 | 6113030 | 1345471 | 10.4 | 215318 | 3238772 | 525438 | 11.6 |
| 41.289 | β-Damascenone | 83361 | 846701 | 407280 | 7.5 | 51876 | 567721 | 262097 | 6.1 | 45312 | 1087402 | 360737 | 10.4 | 25496 | 622584 | 237110 | 20.0 |
| 65.536 | Indole | 25070 | 369627 | 262376 | 11.6 | 71856 | 237262 | 114612 | 8.8 | 44171 | 627043 | 209627 | 9.4 | 26034 | 458449 | 132618 | 16.1 |
|  | **Mean** | **46053** | **1666965** | **261666** |  | **44353** | **1094725** | **229349** |  | **43291** | **2190891** | **223162** |  | **38353** | **1121558** | **172650** |  |
|  | **Acids** |  |  |  |  |  |  |  |  |  |  |  |  |  |  |  |  |
| 24.391 | Acetic acid | 56589 | 378498 | 195145 | 14.1 | 51325 | 249430 | 121062 | 8.5 | 80242 | 416630 | 149573 | 8.6 | 67739 | 1232013 | 129842 | 23.2 |
| 34.910 | 2-methyl butanoic acid | 31655 | 217738 | 76975 | 13.7 | 15395 | 440399 | 43186 | 11.3 | 19196 | 245932 | 102805 | 8.3 | 26930 | 433341 | 117783 | 13.7 |
| 47.364 | Hexanoic acid | 14040 | 102808 | 20738 | 14.4 | 29893 | 117995 | 43740 | 10.2 | 21617 | 76542 | 32866 | 15.5 | 22570 | 210510 | 61270 | 17.5 |
| 51.715 | Octanoic acid | 10419 | 280776 | 26273 | 11.6 | 28787 | 67775 | 39166 | 23.1 | 19356 | 135240 | 40710 | 10.0 | 23460 | 172302 | 59549 | 18.3 |
| 59.845 | Decanoic acid | 16812 | 468106 | 49420 | 13.9 | 37855 | 387028 | 180217 | 14.1 | 2856 | 661621 | 219962 | 10.0 | 85992 | 521272 | 208846 | 23.3 |
| 64.996 | Benzenecarboxylic acid | 59056 | 156632 | 95388 | 10.0 | 34848 | 110795 | 54701 | 9.6 | 13636 | 4829208 | 72214 | 10.3 | 16888 | 264356 | 61699 | 24.4 |
|  | **Mean** | **31428** | **267426** | **77323** |  | **33017** | **228903** | **80345** |  | **26150** | **1060862** | **103021** |  | **40596** | **472299** | **106498** |  |
|  | **Furanic compounds** |  |  |  |  |  |  |  |  |  |  |  |  |  |  |  |  |
| 2.019 | Furan | 81043 | 2277417 | 706292 | 8.1 | 168596 | 1412884 | 661238 | 9.4 | 185407 | 1667357 | 818781 | 8.6 | 419369 | 2115293 | 857008 | 11.3 |
| 2.547 | 2-Methylfuran | 59880 | 4422939 | 306003 | 12.6 | 230881 | 430781 | 287456 | 12.8 | 91312 | 1013850 | 340705 | 10.5 | 163612 | 876383 | 405347 | 18.7 |
| 3.794 | 2,5-Dimethyl-furan | 128108 | 475814 | 247666 | 9.3 | 95912 | 1065607 | 192618 | 10.0 | 52557 | 903996 | 408331 | 5.7 | 106524 | 626124 | 360443 | 14.7 |
| 7.356 | Geraniol oxide | 8947 | 230142 | 68654 | 8.4 | 7279 | 25057 | 21069 | 11.7 | 13968 | 100076 | 39833 | 7.3 | 4991 | 106455 | 61549 | 14.6 |
| 20.314 | 2-Methyl-5-(methylthio)furan | 47978 | 508897 | 142167 | 9.6 | 69782 | 161202 | 106075 | 7.8 | 32478 | 412992 | 111264 | 9.2 | 19465 | 143740 | 80090 | 17.2 |
| 24.964 | Furfural | 31303 | 299015 | 70941 | 11.8 | 16296 | 169291 | 63388 | 8.3 | 32836 | 737181 | 55535 | 8.7 | 12311 | 202585 | 47059 | 13.9 |
| 34.754 | 2-Furanmethanol | 15560 | 68167 | 39151 | 13.3 | 12054 | 51183 | 31719 | 12.4 | 15804 | 109090 | 53420 | 9.9 | 8355 | 94467 | 33786 | 18.1 |
|  | **Mean** | **53259** | **1183199** | **225839** |  | **85828** | **473715** | **194795** |  | **60623** | **706363** | **261124** |  | **104946** | **595006** | **263612** |  |
|  | **Sulfur compounds** |  |  |  |  |  |  |  |  |  |  |  |  |  |  |  |  |
| 1.650 | Methanethiol | 332464 | 1236873 | 806953 | 7.6 | 256844 | 710712 | 393583 | 13.6 | 283024 | 1588958 | 793666 | 9.8 | 95694 | 775948 | 515980 | 17.5 |
| 5.974 | Dimethyl disulfide | 186627 | 9778730 | 2442510 | 7.9 | 973658 | 6416720 | 3505031 | 7.6 | 615927 | 10075468 | 3226247 | 7.9 | 1288974 | 20918014 | 5993939 | 16.6 |
| 16.640 | 2-Methoxythiophene | 59880 | 612164 | 247756 | 9.4 | 166198 | 437645 | 298113 | 15.0 | 158343 | 732175 | 417223 | 9.8 | 125904 | 453897 | 221765 | 18.6 |
| 19.906 | Dimethyl trisulfide | 41561 | 448792 | 152522 | 16.8 | 74166 | 668950 | 132199 | 19.9 | 30127 | 507328 | 180272 | 12.0 | 32488 | 377111 | 123518 | 20.2 |
|  | **Mean** | **155133** | **3019140** | **912435** |  | **367717** | **2058507** | **1082231** |  | **271855** | **3225982** | **1154352** |  | **385765** | **5631242** | **1713800** |  |
|  | **Volatile phenols** |  |  |  |  |  |  |  |  |  |  |  |  |  |  |  |  |
| 43.357 | 2-Methoxyphenol | 38172 | 845615 | 106562 | 10.0 | 27632 | 209200 | 39223 | 10.2 | 32870 | 138149 | 58041 | 11.5 | 31607 | 141277 | 47675 | 16.9 |
| 49.530 | Phenol | 323050 | 981994 | 560972 | 10.1 | 270204 | 646862 | 485137 | 11.4 | 305882 | 2538934 | 627903 | 8.2 | 241116 | 762728 | 410839 | 13.3 |
| 50.903 | Eugenol | 3271 | 799534 | 128786 | 15.3 | NF | NF | NF | - | 8193 | 38350 | 13862 | 10.5 | NF | NF | NF | - |
| 52.608 | 4-Methyl-phenol | 168182 | 18029869 | 1724307 | 12.2 | 139974 | 2997114 | 1517601 | 12.1 | 121595 | 6047040 | 1720270 | 8.6 | 83482 | 5149207 | 857140 | 14.7 |
| 56.904 | 2-Methoxy-4-vinylphenol | 83234 | 203456 | 149040 | 7.5 | 97206 | 108705 | 102956 | 6.7 | 37527 | 214904 | 105022 | 8.3 | 42314 | 209381 | 71998 | 13.3 |
| 60.404 | *p*-tert-butyl-phenol | 785236 | 2309849 | 1589604 | 10.4 | 787204 | 2352610 | 1307262 | 7.3 | 812581 | 2547511 | 1653945 | 7.5 | 816824 | 2565200 | 1390624 | 12.5 |
| 61.590 | 2,4-bis(1,1-dimethylethyl)-phenol | 54622 | 2167900 | 1092669 | 8.8 | 319245 | 1887831 | 1104350 | 7.4 | 64461 | 1831679 | 1242646 | 5.6 | 580650 | 2134308 | 1148274 | 17.2 |
|  | **Mean** | **207967** | **3619745** | **764563** |  | **273577** | **1367053** | **759421** |  | **197587** | **1908081** | **774527** |  | **299332** | **1827017** | **654425** |  |
|  | **Esters** |  |  |  |  |  |  |  |  |  |  |  |  |  |  |  |  |
| 2.846 | Ethyl acetate | 30158 | 72187 | 37650 | 10.2 | 22649 | 81776 | 27975 | 11.0 | 26454 | 273861 | 46690 | 4.8 | 15378 | 759144 | 43270 | 10.9 |
|  | **Mean** | **30158** | **72187** | **37650** |  | **22649** | **81776** | **27975** |  | **26454** | **273861** | **46690** |  | **15378** | **759144** | **43270** |  |
|  | **Higher alcohols** |  |  |  |  |  |  |  |  |  |  |  |  |  |  |  |  |
| 29.656 | 1-Octanol | 17249 | 94081 | 60272 | 12.0 | 39497 | 240892 | 112660 | 8.1 | 23811 | 370082 | 117544 | 10.6 | 29936 | 170474 | 89979 | 12.9 |
| 39.292 | 1-Decanol | 24060 | 41163 | 40865 | 15.2 | NF | NF | NF | - | 19781 | 54871 | 37137 | 10.5 | NF | NF | NF | - |
|  | **Mean** | **20655** | **67622** | **50569** |  | **39497** | **240892** | **112660** |  | **21796** | **212476** | **77341** |  | **29936** | **170474** | **89979** |  |
|  | **Naphthalene derivatives** |  |  |  |  |  |  |  |  |  |  |  |  |  |  |  |  |
| 21.667 | 1,2,3,4-tetrahydro-1,5,7-trimethyl-naphthalene | 17060 | 113473 | 49527 | 8.5 | 3135 | 128625 | 29335 | 6.2 | 9007 | 233185 | 33881 | 10.5 | 9509 | 98088 | 21792 | 20.7 |
| 28.434 | 1,2,3,4-tetrahydro-1,1,6-trimethyl-naphthalene | 4546 | 235568 | 62432 | 7.5 | 10111 | 338732 | 52344 | 8.4 | 10443 | 671723 | 68953 | 14.1 | 7331 | 150502 | 35056 | 22.0 |
| 37.605 | 1,2-Dihydro-1,1,6-trimethyl-naphthalene | 80479 | 5674547 | 1537778 | 11.6 | 290360 | 1443709 | 686436 | 6.2 | 343461 | 2653303 | 1030683 | 14.0 | 52485 | 1757287 | 669024 | 18.9 |
| 48.537 | 2,6-Dimethyl-naphthalene | 8279 | 91251 | 35533 | 9.5 | 7836 | 33754 | 20389 | 5.3 | 13794 | 82432 | 29818 | 12.0 | 12980 | 70818 | 28080 | 10.6 |
| 55.422 | 1,4,5-trimethyl-naphthalene | 30353 | 175390 | 62880 | 16.8 | 11729 | 104334 | 47047 | 8.5 | 15954 | 92687 | 33889 | 10.5 | 9129 | 73932 | 30098 | 13.4 |
|  | **Mean** | **28143** | **1258046** | **349630** |  | **64634** | **409831** | **167110** |  | **78532** | **746666** | **239445** |  | **18287** | **430125** | **156810** |  |
|  | **Other compounds** |  |  |  |  |  |  |  |  |  |  |  |  |  |  |  |  |
| 36.396 | 4-(1-methylethyl)-1-cyclohexene-4-carboxaldehyde | 34791 | 873329 | 37724 | 14.8 | 49730 | 147984 | 91905 | 7.0 | 8084 | 304331 | 74548 | 8.3 | 25949 | 56161 | 33832 | 12.1 |
| 45.221 | 2,7-Dimethyl-quinoline | 22633 | 1291810 | 175956 | 7.8 | 60145 | 182719 | 121744 | 11.6 | 11722 | 240228 | 57095 | 9.1 | 16050 | 346062 | 64055 | 18.1 |
|  | **Mean** | **28712** | **1082570** | **106840** |  | **54937** | **165352** | **106825** |  | **9903** | **272280** | **65821** |  | **20999** | **201112** | **48943** |  |
